# Male-Specific Long Noncoding RNA *TTTY15* Inhibits Non-Small Cell Lung Cancer Proliferation and Metastasis via TBX4

**DOI:** 10.3390/ijms20143473

**Published:** 2019-07-15

**Authors:** I-Lu Lai, Ya-Sian Chang, Wen-Ling Chan, Ya-Ting Lee, Ju-Chen Yen, Chin-An Yang, Shih-Ya Hung, Jan-Gowth Chang

**Affiliations:** 1 Epigenome Research Center, China Medical University Hospital, Taichung 404, Taiwan; 2 Department of Laboratory Medicine, China Medical University Hospital, Taichung 404, Taiwan; 3 Department of Bioinformatics and Medical Engineering, Asia University, Taichung 413, Taiwan; 4 Division of General Pediatrics, Children’s Hospital of China Medical University, Taichung 404, Taiwan; 5 College of Medicine, China Medical University, Taichung 404, Taiwan; 6 Division of Laboratory Medicine, China Medical University Hsinchu Hospital, Hsinchu 302, Taiwan; 7 Graduate Institute of Acupuncture Science, China Medical University, Taichung 404, Taiwan

**Keywords:** *TTTY15*, lncRNA, non-small cell lung cancer, TBX4, methylation, DNMT

## Abstract

Gender affects cancer susceptibility. Currently, there are only a few studies on Y chromosome-linked long noncoding RNAs (lncRNAs), and the potential association between lncRNAs and cancers in males has not been fully elucidated. Here, we examined the expression of testis-specific transcript Y-linked 15 (*TTTY15*) in 37 males with non-small cell lung cancer (NSCLC), and performed circular chromosome conformation capture with next-generation sequencing to determine the genomic interaction regions of the *TTTY15* gene. Our results showed that the expression levels of *TTTY15* were lower in NSCLC tissues. Lower *TTTY15* expression levels were associated with Tumor-Node-Metastasis (TNM) stage. A *TTTY15* knockdown promoted malignant transformation of NSCLC cells. Based on the bioinformatics analysis of circular chromosome conformation capture data, we found that T-box transcription factor 4 (TBX4) may be a potential target gene of *TTTY15*. The RNA immunoprecipitation and chromatin immunoprecipitation results showed that *TTTY15* may interact with DNA (cytosine-5)-methyltransferase 3A (DNMT3A), and the *TTTY15* knockdown increased the binding of DNMT3A to the TBX4 promoter. We concluded that low *TTTY15* expression correlates with worse prognosis among patients with NSCLC. *TTTY15* promotes TBX4 expression via DNMT3A-mediated regulation. The identification of lncRNAs encoded by male-specific genes may help to identify potential targets for NSCLC therapy.

## 1. Introduction

Lung cancer is one of the most frequently diagnosed malignant tumors and remains the leading cause of cancer-related deaths worldwide [1,2]. According to the biological characteristics, there are two main types of lung cancer: non-small cell lung cancer (NSCLC; approximately 85% of lung cancer cases) and small cell lung cancer (SCLC) [3,4]. Since diagnosis is usually made at advanced stages of the disease, the five year overall survival rate among NSCLC patients is under 20% [5,6]. In addition, the overall survival rate is two-fold higher among women than among men. Nonetheless, there is no conclusive evidence to explain the difference in the survival rate between the genders.

The protein-coding genes of the X and Y chromosomes have been relatively well characterized. Some studies have shown that the dysregulation of X inactivation and the loss of the Y chromosome are involved in several types of cancers. Human Y chromosome deletions and rearrangements are associated with various cancers, including prostate cancer [7,8], bladder cancer [9], male sex cord stromal tumors, lung cancer [10,11], and esophageal carcinoma [12], indicating the presence of oncogenes and tumor suppressor genes on this chromosome. Nevertheless, the chromosomal regions responsible for the production of noncoding RNAs are not well known.

Long noncoding RNAs (lncRNAs) are transcribed from thousands of loci in the mammalian genome and play a wide range of roles in gene regulation and other cellular processes, including mRNA splicing, RNA decay, translation, and chromatin remodeling. In addition to contributing to a normal physiology, lncRNA expression and function have been linked to the initiation and progression of cancer. Differences in cancer susceptibility are consistently associated with gender in cancer epidemiology [13]. Men have worse prognoses and a higher mortality rate than women do. Nevertheless, so far, the sex-related lncRNAs are poorly characterized and the potential link between these lncRNAs and cancer has not been studied.

The human Y chromosome can be divided into three regions: (a) male-specific regions of the Y chromosome (MSY), (b) pseudoautosomal regions (PAR1 and PAR2), and (c) heterochromatin in the Yq area [14]. The Y chromosome contains more than 200 specific genes that are important for male sex determination, germ cell differentiation, and masculinization of various tissues [15]. On the other hand, the cellular function of many lncRNAs encoded in MSY has yet to be elucidated. Recently, a study revealed that an intergenic Y-linked lncRNA named lnc-KDM5D-4 (lysine demethylase 5D) is associated with fatty liver, atherosclerosis, and coronary artery disease in men [16]. There is currently only a limited amount of data available regarding the association between Y chromosome-linked lncRNAs and human phenotypes. Moreover, the regulatory mechanisms of these lncRNAs remain to be studied.

Testis-specific transcript Y-linked 15 (*TTTY15*, 5263 bp) is encoded in the chromosome region Yq11.21 of MSY. Some studies have shown that a fusion of the *TTTY15* gene with USP9Y (*TTTY15*–USP9Y) predicts prostate biopsy results [17,18] in multiple cases of hepatocellular carcinoma, lung cancer, and prostate cancer, suggesting that *TTTY15*-USP9Y fusion is a potential driver of carcinogenesis. Nevertheless, the function of *TTTY15* itself in cells remains unknown. In this study, we investigated the mechanism of action of *TTTY15* in the progression of NSCLC. We found that *TTTY15* was significantly downregulated in NSCLC tissue samples compared to adjacent noncancerous tissues. Furthermore, decreased expression of *TTTY15* was associated with poor prognosis among patients with NSCLC. Our data also indicate that a knockdown of *TTTY15* promotes cell proliferation, cell cycle progression, migration, and invasion. In addition, investigation of the mechanism revealed that *TTTY15* can target and affect T-box transcription factor 4 (TBX4) expression via DNA (cytosine-5)-methyltransferase 3A (DNMT3A)-mediated regulation. Our results show for the first time that *TTTY15* participates in NSCLC progression.

## 2. Results

### 2.1. TTTY15 Expression is Low in NSCLC

**Figure 1:**
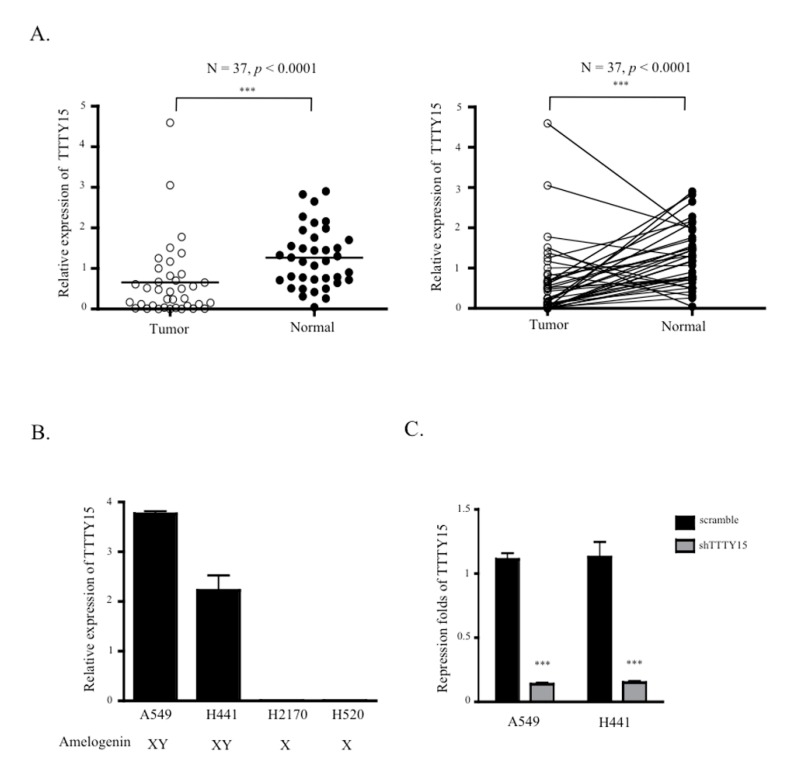
Relative expression and clinical significance of testis-specific transcript Y-linked 15 (*TTTY15*) in non-small cell lung cancer (NSCLC). (**A**) *TTTY15* was found to be downregulated in human NSCLC compared to the adjacent normal tissue (left). The paired data for the *TTTY15* expression in tumor and normal tissue is shown on the right (*n* = 37 for each group). (**B**) The relative expression levels of *TTTY15* in NSCLC cell lines were validated by quantitative reverse-transcription PCR (qRT-PCR). (**C**) The efficiency of the *TTTY15* knockdown in A549 and H441 cells was determined by qRT-PCR. Statistical analysis was based on Student’s *t*-test (*n* = 3); *** *p* < 0.001.

To determine whether *TTTY15* plays a role in NSCLC, we tested the expression of *TTTY15* in 37 pairs of NSCLC tissue samples and their paired nontumor tissue samples. The data suggested that *TTTY15* expression in tumor tissue was significantly lower than that in the matching nontumor tissue (mean delta cycle threshold (dCT) of tumors vs. normal tissue samples: 0.65 vs. 1.26, *p* < 0.0001; Figure 1A). To investigate the correlation between *TTTY15* expression and the clinical features of the patients, NSCLC tissues were categorized into low *TTTY15* expression group (*n* = 27) and high *TTTY15* expression group (*n* = 10) on the basis of the mean value of *TTTY15* expression levels. We found that the expression level of *TTTY15* correlated with Tumor-Node-Metastasis (TNM) stage but not age or tumor size (Table 1). These results indicate that *TTTY15* may participate in NSCLC tumorigenesis. Next, we investigated *TTTY15* expression in NSCLC cell lines, including adenocarcinoma (A549, H441) and squamous cell lung cancer (H2170, H520) cell lines. The amelogenin of squamous cell lung cancer cell lines, H2170 and H520, was only of the “X” type, and these cells did not express *TTTY15* (Figure 1B). Thus, we chose A549 and H441 cells, which express amelogenin X and Y, for the following experiments.

### 2.2. A Knockdown of TTTY15 Increased Lung Cancer Cells Proliferation and Cell Cycle Progression

**Figure 2:**
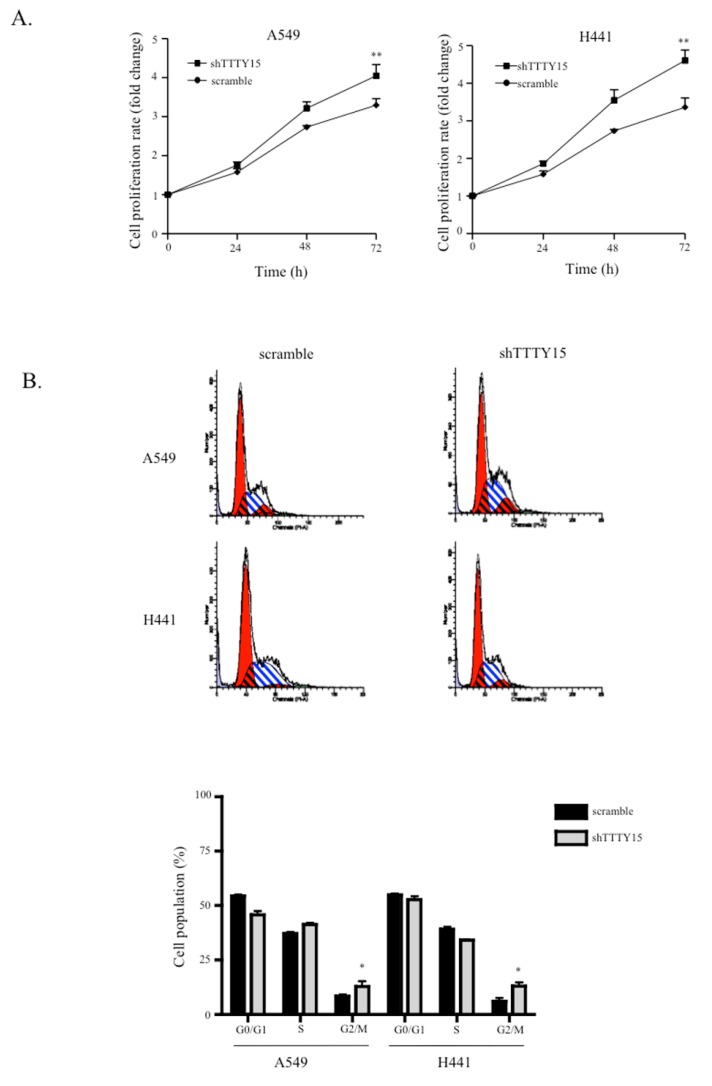
The *TTTY15* knockdown increases cell proliferation and cell cycle progression. (**A**) The 3-(4,5-dimethylthiazol-2-yl)-2,5-diphenyltetrazolium bromide (MTT) assay shows that the *TTTY15* knockdown increased the cell proliferation of A549 and H441 cells. (**B**) Representative images and results of quantification of the flow cytometry data. The cell cycle analyses revealed that *TTTY15* influences A549 and H441 cell proliferation by regulating their cell cycle. The bar chart shows the percentage of cells in G0/G1 phases, in the S phase, and in G2/M phases. Data are presented as the mean ± standard deviation (SD) of three independent experiments. All experiments were conducted in triplicate; * *p* < 0.05, ** *p* < 0.01. Statistical analysis was conducted by Student’s *t*-test (*n* = 3).

To determine the biological functions of *TTTY15* in NSCLC cells, we first constructed stable knockdown cell lines sh*TTTY15*-A549 and sh*TTTY15*-H441 cells. The qRT-PCR data showed that *TTTY15* was repressed by ~90% in the two cell lines (Figure 1C). We then performed a 3-(4,5-dimethylthiazol-2-yl)-2,5-diphenyltetrazolium bromide (MTT) assay to test whether *TTTY15* participated in cancer cell proliferation. The results showed that the knockdown of *TTTY15* in NSCLC cell lines increased cell proliferation (Figure 2A). Next, to examine whether the effect of *TTTY15* on cell proliferation reflects cell cycle progression, we performed a flow cytometry experiment to determine the cell cycle distribution. The results revealed that the knockdown of *TTTY15* increased the prevalence of the G2–M transition among A549 and H441 cells (Figure 2B). These data indicated that *TTTY15* may inhibit the proliferation of NSCLC cells.

### 2.3. The Knockdown of TTTY15 Increased NSCLC Cell Migration and Invasion

**Figure 3:**
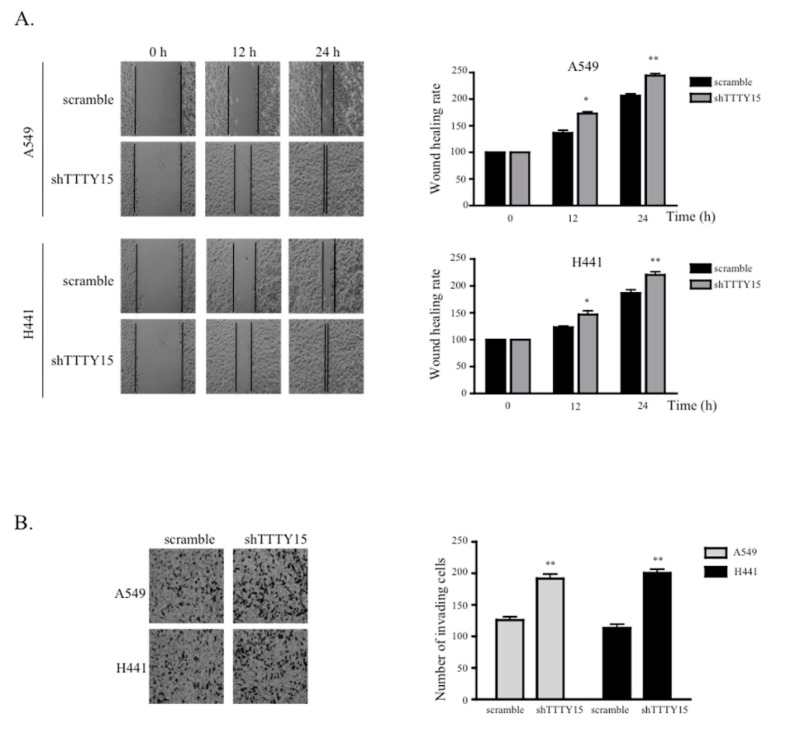
The *TTTY15* knockdown increases cell migration and invasion. (**A**) Wound scratch assays were performed to analyze the migration efficiency of *TTTY15* knockdown A549 and H441 cells compared to the scramble cells. The statistical analysis of the inhibition rates of A549 and H441 cells was performed at 12 and 24 h, respectively. Magnification, ×200. (**B**) The Transwell invasion assay was performed to determine the invasion ability of *TTTY15* knockdown A549 and H441 cells compared to the scramble cells. Data are presented as the mean ± SD; * *p* < 0.05, ** *p* < 0.01. Statistical analysis was conducted by Student’s *t*-test (*n* = 3).

To investigate the effects of *TTTY15* on the migration of A549 and H441 cells, we performed a wound healing scratch assay. The data revealed that the proportion of the recovered region increased for the *TTTY15* knockdown A549 and H441 cells as compared to the scramble cells (Figure 3A). Furthermore, we performed a Transwell invasion assay to evaluate the influence of *TTTY15* on the invasiveness of NSCLC cells. The results indicated that the knockdown of *TTTY15* significantly increased cell invasion as compared to the scramble cells (Figure 3B). These data meant that the knockdown of *TTTY15* may promote cell migration and the invasive motility of NSCLC cells.

### 2.4. TBX4 Is a Potential Target Gene of TTTY15 and Is Positively Regulated by TTTY15

**Figure 4:**
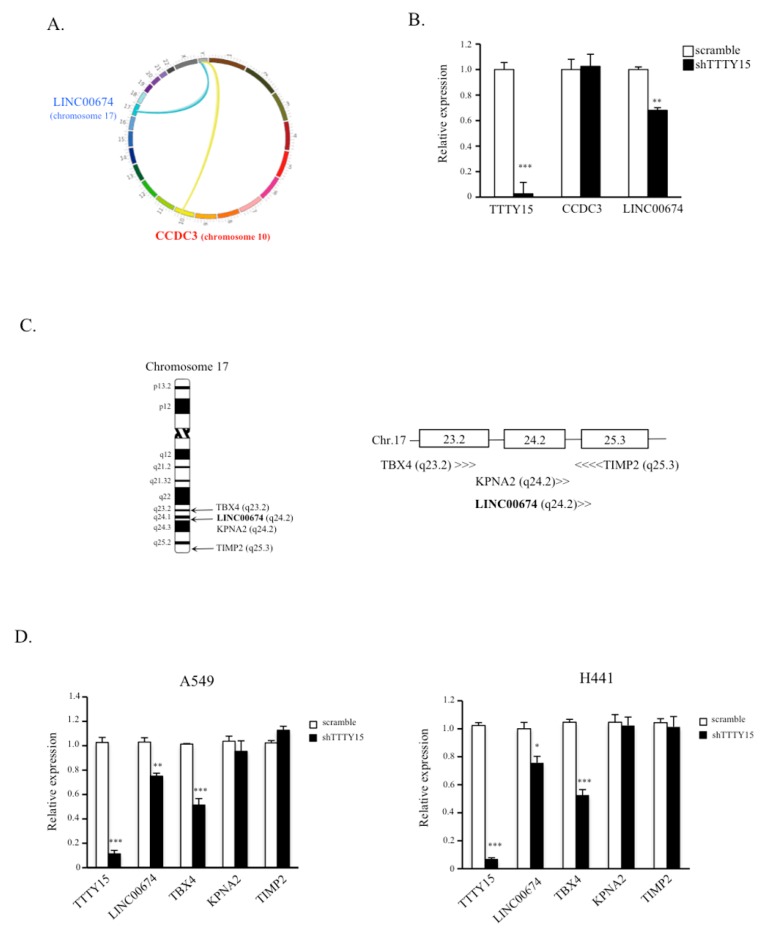
T-box transcription factor 4 (TBX4) is a potential target gene of *TTTY15* and its expression may be regulated by *TTTY15*. (**A**) The *TTTY15* interaction map. (**B**) Comparison of the expression levels of *CCCD3* and *LINC00674* between *TTTY15*-knockdown cells and scramble cells. (**C**) The gene locus on chromosome 17 and a schema of *TTTY15*-targeted *LINC00674* (bold) and the protein-coding genes near *LINC00674*. (**D**) Expression levels of protein-coding genes located near the *LINC00674* gene in *TTTY15* knockdown cells and scramble cells. Data are presented as the mean ± SD; * *p* < 0.05, ** *p* < 0.01, *** *p* < 0.001. Statistical analysis was conducted by Student’s *t*-test (*n* = 3).

Recent studies showed that lncRNAs may modulate large-scale gene expression programs by interacting with chromatin at several thousand different locations across multiple chromosomes. To further explore *TTTY15*-driven regulation of cellular processes, we applied circular chromosome conformation capture (4C) coupled with next-generation sequencing to identify the *TTTY15*-interaction regions in A549 cells. The results indicated that *TTTY15* might interact with *KCNT*2, *WDR26*, *CCDC3*, and *LINC00674* (Table S1). After confirmation of the targeted genes using *CviQ* I and *Sac* I restriction enzyme sites, only *CCDC3* and *LINC00674* fitted the criteria. The trans-interactions between *TTTY15* (located on the Y chromosome) with genes *CCDC3* (chromosome 10) and *LINC00674* (chromosome 17) are presented in Figure 4A. To determine the effects of *TTTY15* on these two target genes, we determined the expression levels of *LINC00674* and *CCDC3* in A549 and H441 cells. The data suggested that the knockdown of *TTTY15* decreased the expression levels of *LINC00674* but not *CCDC3* (Figure 4B), indicating that *TTTY15* may target and regulate *LINC00674* expression. *LINC00674*, located in chromosomal region 17q23, is a long noncoding RNA gene, however, the function of *LINC00674* is still unknown. Since lncRNA may regulate expression of genes on a large scale, we investigated whether *TTTY15* could interact with this chromatin region and regulate the transcription of protein-coding genes located near *LINC00674*, including *TBX4*, *KPNA2*, and *TIMP2* (Figure 4C). The results revealed that the knockdown of *TTTY15* dramatically decreased the expression of TBX4 in A549 and H441 cells (Figure 4D). The spatial proximity of *TTTY15* and *LINC00674* chromatin segments was revealed through *TTTY15* 4C-sequencing and bioinformatic analysis. Moreover, *TTTY15* was found to regulate the expression of TBX4 located near *LINC00674*.

### 2.5. A Knockdown of TTTY15-Targeted TBX4 Increased Lung Cancer Cell Migration and Invasion

**Figure 5:**
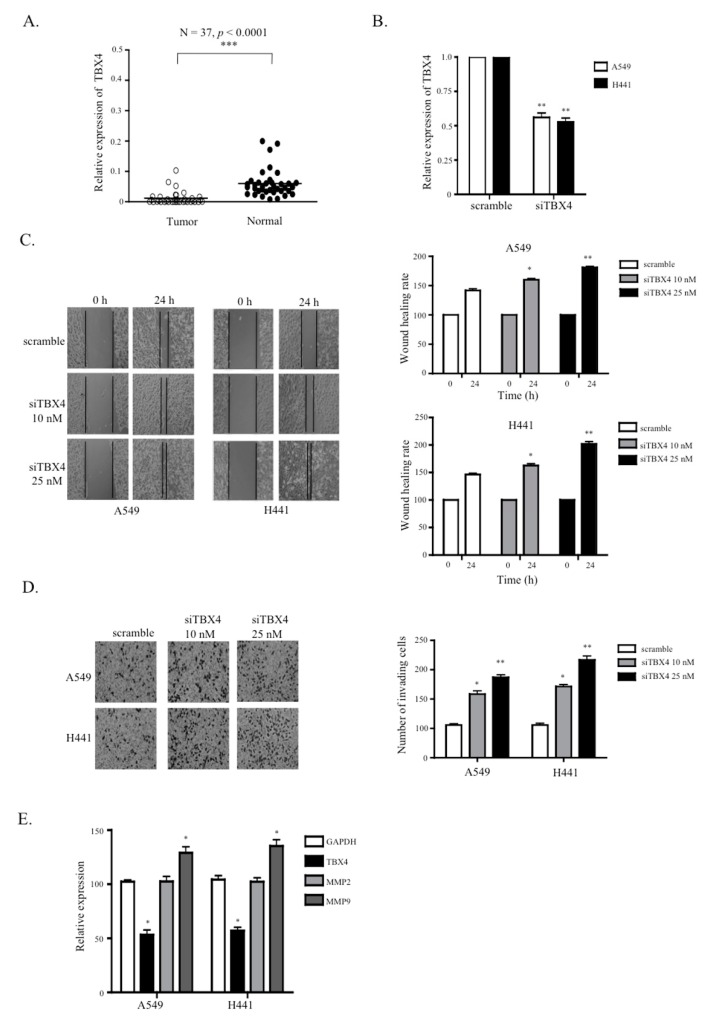
TBX4 was found to be downregulated in NSCLC, and the knockdown of TBX4 increased cell migration and invasion. (**A**) TBX4 is under-expressed in human NSCLC compared to the adjacent normal tissue (*n* = 37 for each group). (**B**) qRT-PCR was carried out to analyze the TBX4 knockdown efficiency of TBX4-specific small interfering RNA (siTBX4) in A549 and H441 cells. (**C**) Wound scratch assays were performed to analyze the migration abilities of TBX4 knockdown A549 and H441 cells compared to the scramble cells. The statistical analysis of the inhibition rates of A549 and H441 cells were performed at 18 h. Magnification, ×200. (**D**) Transwell invasion assays were performed to determine the invasion ability of TBX4 knockdown A549 and H441 cells compared to the scramble cells. (**E**) The knockdown of TBX4 increased the expression levels of matrix metallopeptidase (MMP) 9. Data are presented as the mean ± SD; * *p* < 0.05, ** *p* < 0.01. Statistical analysis was conducted by Student’s *t*-test (*n* = 3).

Since the knockdown of *TTTY15* inhibited TBX4 expression, we next elucidated the involvement of TBX4 in NSCLC. We determined the expression levels of TBX4 in NSCLC tissue samples and paired nontumor tissue samples. The data suggested that TBX4 was downregulated in tumor tissue samples (mean dCT of tumors vs. normal tissues: 0.01 vs. 0.06, *p* < 0.0001; Figure 5A). We also investigated the correlation between TBX4 expression and the clinical features of the patients. NSCLC tissues were categorized into low TBX4 expression group (*n* = 28) and high TBX4 expression group (*n* = 9) on the basis of the mean value of TBX4 expression levels. We found that the expression level of TBX4 was related to TNM stage but not age or tumor size (Table S2). To further examine the role of TBX4 in NSCLC, a TBX4-specific small interfering RNA (siRNA called siTBX4) was designed and transfected into A549 and H441 cells. As depicted in Figure 5B, the cells transfected with siTBX4 showed significantly lower mRNA expression levels of TBX4 as compared to scramble control in both cell lines (*p* < 0.01; Figure 5B). In addition, the wound healing scratch assay and invasion assay suggested that the knockdown of TBX4 decreased the cell migration and invasion abilities (Figure 5C,D). We next tested whether TBX4 regulates the expression of matrix metallopeptidases (MMPs). The qRT-PCR results revealed that the TBX4 knockdown significantly increased the expression of MMP9 but not MMP2 (Figure 5E). These results indicated that the knockdown of TBX4 may promote NSCLC cell invasion and migration.

### 2.6. TTTY15 Is Associated with DNMT3A and Decreases Its Binding to the TBX4 Promoter

**Figure 6:**
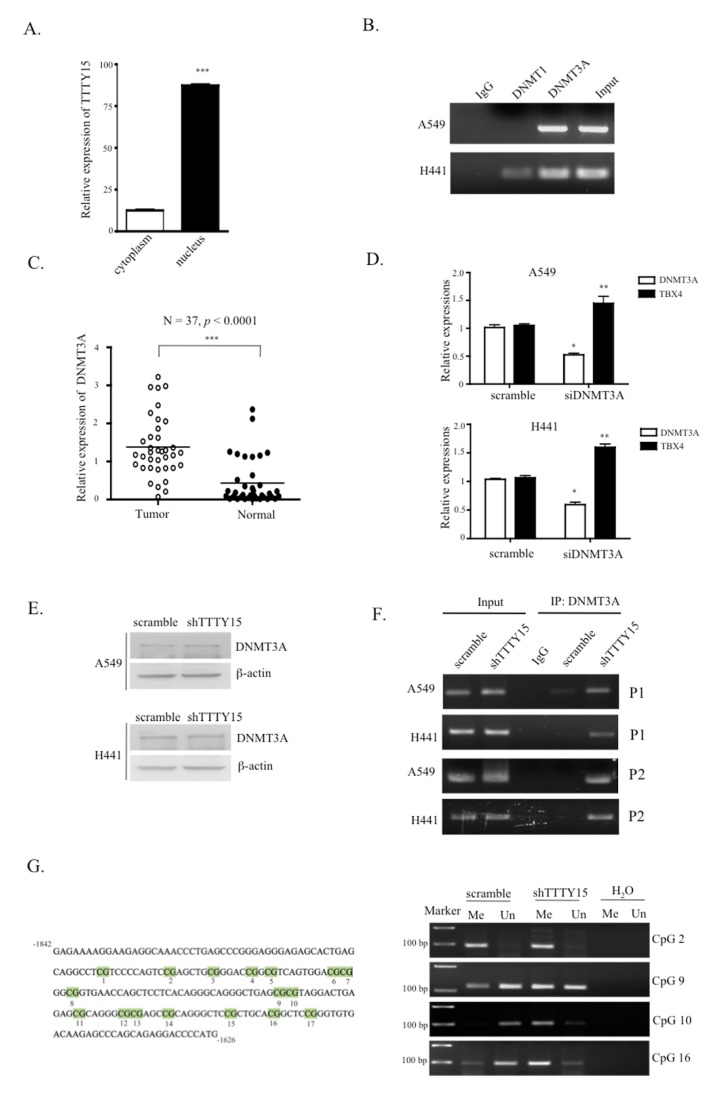
*TTTY15* interacts with DNA methyltransferases (DNMT) 3A and decreases the binding of DNMT3A to the TBX4 promoter. (**A**) Cell fractionation assays were performed to determine the distribution of *TTTY15* expression between the cell cytoplasm and nucleus. (**B**) *TTTY15* RNA coimmunoprecipitated with DNMT1, DNMT3A, or IgG was quantified by qRT-PCR. PCR products were then loaded onto a 3% agarose gel for confirmation. (**C**) DNMT3A turned out to be upregulated in human NSCLC samples compared to the adjacent normal tissue samples (n = 37 for each group). (**D**) The knockdown of DNMT3A increased the expression levels of TBX4 in A549 and H441 cells. (**E**) The knockdown of *TTTY15* did not affect the protein expression levels of DNMT3A. (**F**) Chromatin immunoprecipitation (ChIP)-qPCR was performed to quantify the binding of DNMT3A to the TBX4 promoter (P1: −1734 to −1626 bp; P2: −740 to −672 bp). (**G**) Methylation-specific PCR was performed to analyze the methylation status on the CpG islands of the TBX4 promoter in A549/scramble and A549/sh*TTTY15* (Me: methylated; Un: unmethylated; H_2_O was used as a negative control). Data are presented as the mean ± SD; * *p* < 0.05, ** *p* < 0.01, *** *p* < 0.001. Statistical analysis was conducted by Student’s *t*-test (*n* = 3).

To investigate the potential mechanism by which *TTTY15* regulates TBX4 expression in NSCLC, we analyzed the distribution of *TTTY15* in cells. The cell fractional data indicated that *TTTY15* was mainly localized in the nucleus (Figure 6A). Since other studies suggest that TBX4 is downregulated and hypermethylated in lung cancer-associated fibroblasts [19] and cancers, we speculated that *TTTY15* may participate in the methylation status of the TBX4 promoter. We performed an RNA immunoprecipitation assay to determine whether there is a physical interaction between the DNA methyltransferases (DNMTs) and *TTTY15*. The results revealed that *TTTY15* interacts with DNMT3A but not DNMT1 (Figure 6B). Therefore, we evaluated the expression level of DNMT3A in NSCLC. The results showed that the expression of DNMT3A in tumor tissue was higher than that in the nontumor tissue (mean dCT of tumors vs. normal tissues: 1.37 vs. 0.43, *p* < 0.0001; Figure 6C). We also investigated the correlation between DNMT3A expression and the clinical features of the patients. We found that the expression of DNMT3A correlated TNM stage but not age or tumor size (Table S2). To test whether DNMT3A regulates the expression of TBX4 in NSCLC, DNMT3A was knocked down in A549 and H441 cells. The data revealed that the knockdown of DNMT3A increased the expression of TBX4 (Figure 6D). To elucidate the mechanisms of regulatory action of *TTTY15*, DNMT3A, and TBX4 in NSCLC, we performed Western blotting and chromatin immunoprecipitation (ChIP) on A549 and H441 cell lysates. The results indicated that the knockdown of *TTTY15* did not affect DNMT3A protein expression (Figure 6E) but increased the binding ability of DNMT3A to the TBX4 promoter (Figure 6F). We then performed methylation-specific PCR to verify the CpG methylation status. The results revealed that knockdown of *TTTY15* increased the methylated status in CpG islands (Figure 6G). Next, to determine whether the *TTTY15*–DNMT3A–TBX4 axis is involved in NSCLC, we knocked down DNMT3A and checked whether the cell migration and invasion abilities increased by sh*TTTY15* would be attenuated. The findings showed that the knockdown of DNMT3A indeed inhibited the cell migration and invasion in A549/sh*TTTY15* and H441/sh*TTTY15* cells (Figure S1).

## 3. Discussion

Recent studies suggest that gender affects the risk, incidence, and progression of various human diseases, including cancers [20,21,22,23,24]. The most important genetic difference between men and women is the genes on the sex chromosomes. At present, there is a limited number of studies on Y chromosome-linked lncRNAs in human diseases and cancers because the Y chromosome is often excluded from genomic analysis studies [25,26]. Our study provides the first evidence of the expression of a Y chromosome-linked lncRNA called *TTTY15* in NSCLC. Some authors have reported that ubiquitin specific peptidase 9, Y-linked (USP9Y)–*TTTY15* is expressed in both tumor and nonmalignant samples and can be used to predict the outcome of a prostate biopsy [17,18,27]. The *TTTY15* gene can get fused to >zinc finger DHHC-type containing 2 (ZDHHC2–*TTTY15*), and this fusion is found in patients with acute myeloid leukemia [28]. In addition to forming fusion genes, *TTTY15* (its product) can protect cardiomyocytes from hypoxia-induced cell injury by targeting miR-455-5p and thus regulating Jun dimerization protein 2 (JDP2) expression [29]. *TTTY15* has also been reported to mainly localize in the cytoplasm and promote prostate cancer progression by functioning as an RNA sponge and binding to let-7 [30]. These studies show that *TTTY15* plays important roles in cells. In our study, we found that *TTTY15* was downregulated in lung cancer and was mainly located in the nucleus, upregulating TBX4 expression by targeting DNMT3A. These different findings suggest that *TTTY15* may play a dual part in the cytoplasm and nucleus in various types of cancer. This study is the first to determine the clinical significance and biological function of *TTTY15* in NSCLC, and the results indicate that *TTTY15* is a tumor suppressor in NSCLC.

LncRNAs are long and can form complex secondary and tertiary structures to participate in gene regulation. A growing amount of research is suggesting that lncRNA genes can function as genes in certain situations and can regulate elements located on different chromosomes through processes involving the physical associations between different chromosomes (“trans-interactions”) [31,32,33,34]. Chromosome interactions can promote the silencing and/or activation of genes within the three-dimensional structure of the nuclear matrix [35,36,37]. To elucidate the regulatory mechanism of action of *TTTY15* in the NSCLC, we employed the 4C technique to identify the target regions of *TTTY15* in the genome. The 4C data showed that *TTTY15* targeted *CCCD3* (chromosome 10) and *LINC00674* (chromosome 17). After confirming the knockdown, we found that the knockdown of *TTTY15* dramatically decreased the expression of T-box transcription factor 4 (TBX4). TBX4 is located in chromosomal region 17q23. Its protein product is reported to act as a transcription factor in hind limb growth and to regulate some processes during embryonic development [38,39]. TBX4- and TBX5-deficient mice show severely reduced lung branching during the second trimester, suggesting that TBX4 performs an important function in the regulation of proliferation, migration, and differentiation of mesenchymal cells, in addition to supporting and helping to produce peripheral epithelial cells [40]. In the present study, 4C was used in combination with next-generation sequencing and bioinformatic analysis to investigate the possible interactions of *TTTY15* with other targets in the genome. This approach was effective at identifying the regulation targets of the lncRNA genes.

TBX4 is a mesenchymal transcription factor that drives the accumulation of myofibroblasts and the development of pulmonary fibrosis [41]. Low expression levels of TBX4 indicate a worse prognosis in patients with stage II pancreatic ductal adenocarcinoma (PDAC) [19]. Our results revealed that TBX4 is downregulated in NSCLC and the knockdown of TBX4 increases the NSCLC cell migration and invasion. MMPs are known to degrade proteins in the extracellular matrix and basement membrane, and to promote tumor metastasis [42]. Although the inhibition of the T-box transcription factor Brachyury is shown to downregulate MMP2 and MMP24 in cancer, to date [43], there are no reports on the direct participation of TBX4 in MMP gene expression. Therefore, we conducted experiments here to determine whether there is any correlation between MMP and TBX4. Our data showed that TBX4 silencing increases MMP9 expression in A549 and H441 cells. These results suggest that TBX4 is a tumor suppressor in NSCLC and inhibits the migration and invasiveness of NSCLC by decreasing the activity of MMP9.

In addition to epigenetic control via chromosome modification, lncRNA can also affect gene regulation by its association with DNA methylation. Promoter methylation status is known to be involved in the initiation and progression of tumors. TBX4 is reported to be highly methylated in PDAC and human salivary adenoid cystic carcinoma [19,44]. In mammalian cells, DNMT3A and DNMT3B are known to establish a DNA methylation pattern de novo, while DNMT1 takes part in the maintenance of methylation status during DNA replication [45,46]. One study showed that lncRNA Dum interacts with three DNMTs to mediate and maintain local DNA methylation in the Dppa2 promoter [47]. In the present study, *TTTY15* was found to regulate TBX4 expression via DNA methylation status. Our RNA immunoprecipitation and ChIP data revealed that *TTTY15* can interact with DNMT3A and that the binding of DNMT3A to the promoter of TBX4 was blocked by *TTTY15*. Furthermore, the overexpression of a DNMT in a variety of tumors results in highly methylated and carcinogenically activated genes [48]. Moreover, either DNMT3A or DNMT3B is found in many clinical tumor samples, and the increased expression of DNMT3A was previously reported to participate in the progression of hepatocellular carcinoma [49,50]. Our results also showed that DNMT3A is overexpressed in NSCLC. These findings suggest that *TTTY15* may interact with DNMT3A and decrease the ability of DNMT3A to bind to the TBX4 promoter.

## 4. Materials and Methods

### 4.1. Patients and Sample Collection

Thirty-seven paired NSCLC tissue samples and adjacent nontumor tissue samples from male patients (mean age 65.0, SD ± 9.6) were obtained from the Biobank of China Medical University Hospital (CMUH) between 2006 and 2014. This study was conducted with the approval of the CMUH’s Institutional Research Ethics Committee on 11 December 2014 (CMUH103-REC2-140), according to the Declaration of Helsinki’s guidelines. None of the patients had received radiotherapy or chemotherapy prior to surgical resection. All tumor specimens were snap-frozen and stored in liquid nitrogen until analysis. The clinical and histopathological characteristics of each patient were also recorded. Informed written consent was obtained from all the patients.

### 4.2. Cell Culture and Stable Transfection

Cell lines A549, H441, H2170, and H520 were cultured in in Dulbecco’s Modified Eagle Medium (DMEM) supplemented with 10% fetal bovine serum (10% FBS, Gibco, Gibco, Grand Island, NY, USA), 100 U/mL penicillin, and 100 μg/mL streptomycin in humidified air at 37 °C with 5% of CO_2_. The medium was refreshed every day, and cell passaging was performed once every 3 days using 0.25% trypsin. The synthesis of shRNA (short hairpin RNA) downregulating *TTTY15* was based on the pSUPER RNAi system expression vector (OligoEngine, Seattle, WA, USA). The oligo sequences designed for targeting *TTTY15* were as follows: *TTTY15*-forward: 5′-GATCCCCTTTACAAAGAATTCCAGCTCTGTGGTTCAAGAGACCACAGAGCTGGAATTCTTT GTAAATTTTTA-3′ and *TTTY15*-reverse: 5′-AGCTTAAAAATTTACAAAGAATTCCAGCTCTGTG GTCTCTTGAAGCCACAGAGCTGGAATTCTTTGTAAAGGG-3′. The annealing and ligation procedures were carried out according to the manufacturer’s instructions. The A549 and H441 cells were transfected with the sh*TTTY15* plasmid and selected by means of G418. The efficiency of the *TTTY15* knockdown was determined by qRT-PCR.

### 4.3. SiRNA Transfection

The siRNAs for targeting TBX4 and DNMT3A mRNA were constructed by MDBio Inc. (Taipei, Taiwan). The targeting sequences for TBX4 and DNMT3A were 5′-CCGAUGACCAUCGCUACAA TT-3′ and 5′-CAGUGGUGUGUGUUGAGAATT-3′, respectively. The scrambled ribooligonucleotide served as the negative control (a scrambled %GC matched oligonucleotide). A549 and H441 cells were transfected with various doses of siRNA or a scramble ribooligonucleotide by means of RNAimax Lipofectamine (Invitrogen, Carlsbad, CA, USA), according to the manufacturer’s instructions.

### 4.4. Subcellular Fractionation, Total RNA Extraction, and qRT-PCR Analysis

The nuclear and cytosolic fractions of the A549 and H441 cell lines were fractionated using the PARIS Kit (Life Technologies, Carlsbad, CA, USA), according to the manufacturer’s instructions. Total RNA was extracted with the TRIzol reagent (Life Technologies, Scotland, UK), according to the manufacturer’s instructions. The RNA was quantitated on a nanodrop spectrophotometer (Thermo Scientific, Waltham, MA, USA). RNA was reverse-transcribed into cDNA using the cDNA Reverse Transcriptase Kit (Thermo Fisher Scientific-Applied Biosystems, Waltham, MA, USA). qRT-PCR was performed via a TaqMan assay, where glyceraldehyde 3-phosphate dehydrogenase (GAPDH) served as the internal control for mRNA levels. The relative expression levels of the target genes were calculated as ratios normalized to GAPDH. The primers were synthesized by Genomics BioSci and Tech (Taipei, Taiwan). The following primer sequences were used (5′–3′): *TTTY15*-forward: tgagggagggatgtagctttt; *TTTY15*-reverse: gaagtcaagcaggcaactga; *CCDC3*-forward: tttctagccttttccagttttca; *CCDC3*-reverse: caaacaaggccttctgcac; *LINC00674*-forward: aagctgggctcaagagatcc; *LINC00674*-reverse: tggctgtggtggcttgta; *KPNA2*-forward: tgggccgtgaccaactatac; *KPNA2*-reverse: tgccacagtgaacaaggtaca; *TBX4*-forward: ccatcgctacaagttctgtgac; *TBX4*-reverse: gaatccgggtggacatacag; *TIMP2*-forward: gtgggtccaaggtcctcat; *TIMP2*-reverse: cgaagccccagacacatagt; *DNMT3A*-forward: cctgaagcctcaagagcagt; *DNMT3A*-reverse: tggtctccttctgttctttgc; *MMP2*-forward: ccccaaaacggacaaagag; *MMP2*-reverse: cttcagcacaaacaggttgc; *MMP9*-forward: cgcagacatcgtcatccagt; *MMP9*-reverse: cgcagacatcgtcatccagt. The average value of genes was measured using the 2^−ΔΔ*C*t^ method.

### 4.5. A Cell Proliferation Assay

This assay was conducted to determine whether the *TTTY15* knockdown affected the viability of NSCLC cells. Briefly, *TTTY15* stable knockdown A549 and H441 cells were seeded in 96-well plates (at 5000 cell/well). After 24, 48, and 72 h, cell proliferation and viability were examined in an 3-(4,5-dimethylthiazol-2-yl)-2,5-diphenyltetrazolium bromide (MTT) assay. All the experiments were conducted in triplicate.

### 4.6. Wound Healing Scratch and Transwell Invasion Assays

A wound healing assay was performed to assess the migration ability of cells. A549 and H441 cells were seeded in a 24-well plate as a monolayer at a density of 95–100%. The cell monolayer was gently scratched across the center of the well with a 200 μL plastic pipette tip. The rate of wound closure was observed by imaging by means of an inverted microscope (DMi1; Leica, Wetzlar, Germany). We then used the free software Tscratch to verify and calculate the migration movement of the entire wound area. A cell invasion assay was performed with transwell chambers (8 μm pore size), which were coated with 0.1% gelatin (50 μL/well), in 24-well plates. Approximately 10^5^ either scramble cells or sh*TTTY15* cells were seeded into the upper chamber of the insert. After incubation for 24 h at 37 °C with 5% CO_2_, any cells that had invaded the membrane were stained with a 0.1% crystal violet solution. The cells were counted in three randomly selected visual fields under the inverted microscope (DMi1; Leica). For each experimental group, the assay was performed in triplicate.

### 4.7. Flow Cytometry for Cell Cycle Analysis

Transfected A549 and H441 cells were harvested 48 h after transfection. The cells were fixed in 70% ethanol at −20 °C overnight. The fixed cells were then washed once with phosphate-buffer-saline (PBS) and labeled with propidium iodide (Sigma-Aldrich, St. Louis, MO, USA) in the presence of RNase A (Sigma-Aldrich, St. Louis, MO, USA) and Triton X-100 for 30 min in the dark. The samples were run on a FACSCanto flow cytometer (BD Biosciences, San Diego, CA, USA) and the percentages of the cells within each phase of the cell cycle were analyzed in the ModFit LT software.

### 4.8. Circular Chromosome Conformation Capture

As previously described, 4C was performed [51]. In brief, A549 cells were crosslinked with 1% formaldehyde for 10 min at room temperature to preserve the three-dimensional nuclear architecture. *Sac* I was used to digest the crosslinked chromatin (primary digestion). The digested chromatin was then ligated with the T4 DNA ligase. The digested and ligated chromatin was then de-crosslinked and subjected to the second restriction digestion using *CviQ* I to reduce the size of the fragments. Inverse PCRs were carried out with *TTTY15*-specific primers containing Illumina adapter sequences to amplify the genomic DNA fragments ligated to *TTTY15* (first PCR: forward 5′-tggtgcgatcttgatttactgc-3′, reverse 5′-tgccttttgtctgtatgt gca-3′; nested PCR: forward 5′-tcattcttgttgcccagtctg-3′, reverse 5′-ttcaccttttgttggctccc-3′).

### 4.9. Next-Generation Sequencing and Bioinformatic Analysis of TTTY15 4C-Sequencing Data

The PCR products were purified with the Qiagen Mini-Elute kit (Qiagen, Hilden, Germany). The amplicon was then prepared for sequencing using the TruSeq DNA library preparation kit (Illumina, San Diego, California). After that, 100 ng purified amplicon pools were repaired to generate blunt-ended ligations, according to the TruSeq DNA Sample Preparation protocol. 5′-Phosphorylated DNA and an A-tailing reaction compatible with the adapter ligation strategy were performed. The ligation product was then purified using sample purification beads. In order to enrich the library, an enhanced PCR mix was used for PCR amplification. The size distribution of the library was verified using the High-Sensitivity DNA Kit (Agilent, Technologies, Waldbronn, Germany), and the concentration of the library was quantified with the GeneRead Library Quant Kit (Qiagen, Hilden, Germany). The library was diluted and sequenced with 500 paired-end cycles on the Illumina MiSeq platform by following the standard protocol. For bioinformatic analysis, any known fragments were removed from the sequencing reads as follows. First, the forward and reverse sequencing reads were merged into one sequence, such that the length of the overlapping region was over 20 nucleotides. Next, the bioinformatic software of sequence BLAST was used to identify the location of the known fragments and primers [52]. The known fragments were located in the regions between the primers and the cutting site and the alignment similarity for BLAST was set to 95%. Finally, these primers and known fragments of sequencing reads were removed from the sequences. The remaining region of the sequences was labeled as “unknown fragments”. The Bowtie2 software is an efficient tool for the identification of potential *TTTY15* interaction regions, therefore by aligning sequencing reads against reference sequences [53], we aligned unknown fragments against human genome sequences (Grch38.p2 was employed in the present study) in this software. Subsequently, fourSig was used to identify the potential *TTTY15*-interacting genome regions [54]. After scanning for the interacting regions, fourSig provided two categories of regions that were defined as potential *TTTY15* interaction regions. The regions and their associated genes were listed and annotated according to the known human genomic location. These two interaction regions were used as the targeting candidates for *TTTY15*.

### 4.10. The Western Blot Assay

Whole-cell extracts were prepared from A549 and H441 cells by adding radioimmunoprecipitation assay (RIPA) lysis buffer (150 mM NaCl, 0.1% SDS, 0.5% sodium deoxycholate, 1% NP-40) (Sigma-Aldrich, St. Louis, MO, USA) with complete protease inhibitor cocktails (Sigma). Equal quantities of total protein samples were separated on 10% SDS-PAGE gels and transferred onto poly vinylidene fluoride (PVDF) membranes. The blots were incubated with primary antibody against DNMT3A (ab13888, Abcam, Cambridge, MA, USA) overnight at 4 °C. After second antibody incubation, the electrochemiluminescence (ECL) kit (EMD Millipore, St. Charles, MO, USA) was utilized to visualize the protein signals. β-Actin (Proteintec, Rosemont, IL, USA) served as the internal control.

### 4.11. RNA Immunoprecipitation

This procedure was carried out using ChIP-IT Kit (Active Motif, Carlsbad, CA, USA), according to the manufacturer’s instructions. Briefly, endogenous DNMT1 and DNMT3A complexes from the whole-cell extract were pulled down using anti-DNMT1 (ab13537) or anti-DNMT3A (ab13888, Abcam, Cambridge, MA, USA) antibody-coated beads. The beads were then washed with wash buffer and eluted with elution buffer. The eluted samples were incubated with 0.5 mg/mL protease K to remove proteins. The isolate from the immunoprecipitation product was further validated by qRT-PCR.

### 4.12. Chromatin Immunoprecipitation

The DNA ChIP assay was performed using the ChIP-IT Kit (Active Motif, Carlsbad, CA, USA) according to the manufacturer’s instructions. Briefly, anti-DNMT1 and anti-DNMT3A antibody-coated beads were used to pull down the DNMT1 and DNMT3A complexes from A549 and H441 cells. The beads were washed three times with washing buffer. The beads were then eluted and subjected to reverse crosslinking. The isolate from the IP product was then validated by qRT-PCR. The following primers were designed to amplify the TBX4 promoter region: TBX4 promoter (−749 to −672 bp)-forward: 5′-cagagcctggatcagtcacc-3′, reverse: 5′-tctggcacagacatcctcac-3′ and TBX4 promoter (−1734 to −1626 bp)-forward: 5′-TGAACCAGCTCCTCACAGG-3′, reverse: 5′-CTCTGCTGGGCTCTTG TCAC-3′.

### 4.13. Methylation-Specific PCR

The methylation status of the promoter region on the TBX4 gene (from −1842 to −1626 bp) was analyzed by methylation-specific PCR. Briefly, the genomic DNA of A549 cells was extracted using the QIAamp DNA Mini Kit (Qiagen, Germany) and was then modified with the Zymo EZ DNA Methylation Kit (Zymo Research, Tustin, CA, USA), according to the manufacturer’s instructions. The modified DNA was amplified using a 20 μL mixture including HotStarTag buffer (2.0 mM Mg^2+^, 0.2 mM deoxy-ribonucleoside triphosphate (dNTP), 1 U HotStarTag polymerase (Qiagen, Germany)), 0.2 μM of each primer and 1 μL of template DNA. The amplification program for methylation-specific PCR was as follows: an initial incubation for 10 min at 95 °C, followed by 35 cycles of 95 °C for 30 s, 50 °C for 1 min, 72 °C for 1 s, and a final 5 min incubation at 72 °C. The primer information for methylation-specific PCR was shown in Table S3.

### 4.14. Statistical Analysis

All experimental data from the three independent experiments were analyzed in GraphPad Prism version 5 (GraphPad Software Inc., La Jolla, CA, USA). The results were expressed as the mean ± SD (standard deviation). The associations between the relative *TTTY15* RNA expression levels and the clinical parameters (age, tumor size, lymph node metastasis, and TNM stage) were analyzed by Fisher’s exact test. A Student’s *t*-test was conducted to analyze the differences, where *p* < 0.05 was assumed to indicate a statistically significant difference.

## 5. Conclusions

The Y chromosome contains not only genes involved in sex determination but also genes that participate in the prevention of tumors. This study is the first to provide evidence for the potential involvement of the Y chromosome-linked *TTTY15* in NSCLC. We found that *TTTY15* is downregulated in NSCLC and was associated with lymph node metastasis and the TNM stage of patients with NSCLC. Our study determined a novel target gene of *TTTY15* and revealed the interactions among *TTTY15*, DNMT3A, TBX4, and MMP9 in NSCLC cells. The application of the 4C technique allowed for a genome-wide search for lncRNA target genes and may facilitate further studies on the function of lncRNAs in cancer. Our analysis of the Y chromosome could be conducted to predict the risk of cancer in men.

## Figures and Tables

**Table 1:** *TTTY15* expression and clinical characteristics of NSCLC patients (*n* = 37).

Parameter	*n*	Relative *TTTY15* Expression		*p* Value
		Low (*n* = 27)	High (*n* = 10)	
Age (years)				0.714
≤65	18	14	4	
>65	19	13	6	
Tumor size (maximum diameter)				0.453
≤3 cm	14	9	5	
>3 cm	23	18	5	
Lymph node metastasis				
N1	25	21	4	0.0486
N0	12	6	6	
TNM (Tumor-Node-Metastasis) stage				
I–II	13	6	7	0.0167
III–IV	24	21	3	

## Supplementary Materials

Supplementary materials can be found at https://www.mdpi.com/1422-0067/20/14/3473/s1.
