# Supplementary material for: Male-Specific Long Noncoding RNA TTTY15 Inhibits Non-Small Cell Lung Cancer Proliferation and Metastasis via TBX4
Source: Int J Mol Sci. 2019 Jul 15;20(14):3473. doi: 10.3390/ijms20143473 (PMC6678590; doi:10.3390/ijms20143473)
Supplement: Supplementary file 1 [file ijms-20-03473-s001.pdf]

Table S1. List of *TTTY15* targeted genes detected in 4C.

Gene	Chromosome	Gene description	Gene type	Cutting site check (*CvuQ* I/*Sac* I)
KCNT2	Chromosome 1	Potassium channel, subfamily T, member 2	Protein–coding	False
WDR26	Chromosome 1	WD repeat domain 26	Protein–coding	False
CCDC3	Chromosome 10	Coiled–Coil Domain Containing 3	Protein–coding	True
LINC00674	Chromosome 17	Long intergenic non–protein coding RNA 674	Noncoding RNA	True

Table S2. Clinicopathological features and correlation of TBX4 and DNMT3A expressions in NSCLC patients (n = 37)

		Relative *TBX4* expression			Relative *DNMT3A* expression		
		Low	High		Low	High	
Parameter	N = 37	(N = 28)	(N = 9)	*p* value	(N = 12)	(N = 25)	*p* value
Age (years)				0.714			0.1704
≤65	18	13	5		8	10	
>65	19	15	4		4	15	
Tumor size (maximum diameter)				0.7046			0.4701
≤3 cm	14	10	4		6	8	
>3 cm	23	18	5		6	17	
Lymph node metastasis							
N0	12	6	6	0.0355	7	5	0.0292
N1	25	22	3		5	20	
TNM (Tumor-Node-Metastasis) stage							
I-II	13	6	7	0.0041	8	5	0.0097
III-IV	24	22	2		4	20	

Table S3. Methylation-specific PCR primers for analysis of TBX4 promoter.

CpG islands	Primers	Sequence (5′-3′)	Size (bp)
CpG 2	CpG 2-Me-F	GAGAGTATTGAGTAGGTTTC	93
	CpG 2-Un-F	GAGAGTATTGAGTAGGTT TT	93
	CpG 2-R	CAACCCTACCCTATAAAAAAC	
CpG 9	CpG 9-Me-F	TTATAGGGTAGGGTTGAGC	95
	CpG 9-Un-F	TTATAGGGTAGGGTTGAGT	95
	CpG 9-R	CCTCTACTAAACTCTTATCACA	
CpG 10	CpG 10-Me-F	TATAGGGTAGGGTTGAGYGC	95
	CpG 10-Un-F	TATAGGGTAGGGTTGAGYGT	95
	CpG 10-R	CCTCTACTAAACTCTTATCACA	
CpG 16	CpG 16-Me-F	GTYGTAGGGTTTYGTTGTAC	96
	CpG 16-Un-F	GTYGTAGGGTTTYGTTGTAT	96
	CpG 16-R	CACAAAAAAAAACCATCTCAC	

A.

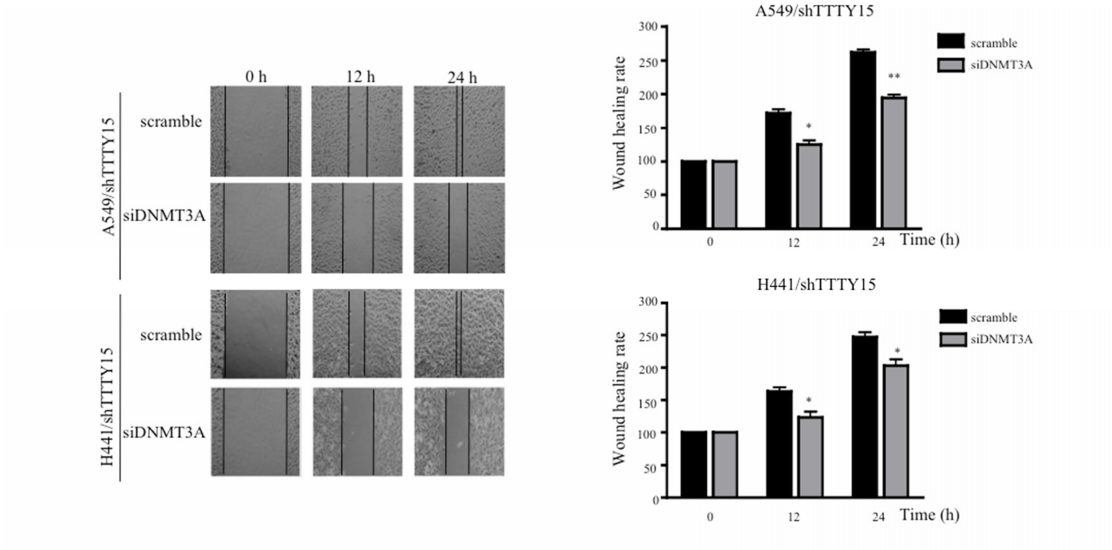

B.

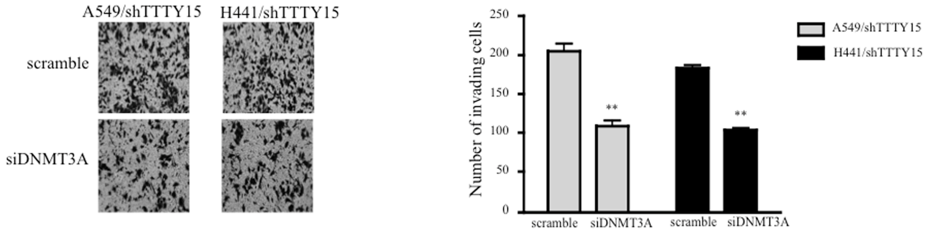

Figure S1. The DNMT3A knockdown rescued the cell migration and invasion abilities in A549/sh*TTTY15* and H441/sh*TTTY15* cells. (A) Wound scratch assays were performed to analyze the migration efficiency of DNMT3A knockdown A549/sh*TTTY15* and H441/sh*TTTY15* cells compared to the scramble cells. Magnification, ×200. (B) Transwell invasion assays were performed to determine the invasion ability of DNMT3A knockdown A549/sh*TTTY15* and H441/sh*TTTY15* cells compared to the scramble cells. Data are presented as the mean ± SD; $*p < 0.05$, $**p < 0.01$. Statistical analysis was conducted by Student's *t*-test (n = 3).
